# Cocaine-Evoked Locomotor Activity Negatively Correlates With the Expression of Neuromedin U Receptor 2 in the Nucleus Accumbens

**DOI:** 10.3389/fnbeh.2018.00271

**Published:** 2018-11-13

**Authors:** James M. Kasper, Ashley E. Smith, Jonathan D. Hommel

**Affiliations:** ^1^Center for Addiction Research, Department of Pharmacology and Toxicology, University of Texas Medical Branch, Galveston, TX, United States; ^2^Department of Neuroscience, Cell Biology and Anatomy, University of Texas Medical Branch, Galveston, TX, United States

**Keywords:** cocaine, neuropeptide, neuromedin U, sensitization, neuromedin U receptor 2

## Abstract

Cocaine use disorder (CUD) is characterized by repeated cycles of drug seeking and drug taking. Currently, there are no available pharmacotherapies to treat CUD, partially due to a lack of a mechanistic understanding of cocaine-evoked alterations in the brain that drive drug-related behaviors. Repeated cocaine use alters expression of numerous genes in addiction-associated areas of the brain and these alterations are in part driven by inter-subject genetic variability. Recent findings have shown the neuropeptide neuromedin U (NMU) and its receptor NMU receptor 2 (NMUR2) decrease drug-related behaviors, but it is unknown if substances of abuse alter NMU or NMUR2 expression. Here, rats were given twice daily saline or cocaine (15 mg/kg, intraperitoneal (IP)) for 5 days and then 7 days with no treatment. All rats were then given a single cocaine treatment and locomotor activity was measured in the acute (non-sensitized) and repeated drug exposure (sensitized) groups. Immediately following locomotor assay, tissue was taken and we demonstrate that accumbal NMUR2 mRNA expression, but not NMU mRNA expression, is negatively correlated with non-sensitized cocaine-evoked locomotor activity, but the correlation is lost following cocaine sensitization. Furthermore, in a separate cohort NMUR2 protein levels also negatively correlated with cocaine-evoked locomotor activity based on immunohistochemical stereology for NMUR2 protein expression. These findings are the first to demonstrate that repeated cocaine exposure causes dysregulated expression of NMUR2 and highlight the deleterious effects of repeated cocaine exposure on neurobiological receptor systems. Restoring the normal function of NMUR2 could be beneficial to the treatment of CUD.

## Introduction

Cocaine use disorder (CUD) is a chronic, relapsing brain disease that is characterized by repeated cycles of drug seeking and drug taking (Koob and Volkow, 2010). There are genetic vulnerability components to CUD (Goldman et al., 2005) and repeated exposure to cocaine alters the expression of genes and proteins in brain regions associated with addiction (Pierce et al., 2018). The effect of repeated cocaine exposure on the brain can be studied using animal models of cocaine sensitization, where repeated cocaine increases future behavioral responses to cocaine (Steketee and Kalivas, 2011). Cocaine-evoked molecular changes in brain regions linked to addiction, such as the nucleus accumbens (NAc), may be associated with behavioral changes associated with repeated exposure to cocaine.

The endogenous neuropeptide neuromedin U (NMU; Kasper et al., 2016; Vallöf et al., 2016, 2017) is highly enriched areas of the brain associated with addiction including the NAc (Domin et al., 1986). The receptor for NMU is the G-protein coupled receptor NMU receptor 2 (NMUR2; Howard et al., 2000; Brighton et al., 2004a,b), which is also expressed in the NAc (Gartlon et al., 2004). Furthermore, NMU has been demonstrated to have therapeutic promise in animal models of substance use disorder (Kasper et al., 2016; Vallöf et al., 2016, 2017). For example, a key finding is that NMU administered directly to the NAc before repeated cocaine prevents cocaine sensitization, whereas NMU administered after repeated cocaine does not alter cocaine sensitization (Kasper et al., 2016). This background framework suggests that repeated cocaine exposure alters the ability of accumbal NMUR2 to respond to NMU and modulate cocaine-evoked behavior. This may be mediated by altered expression of accumbal NMUR2.

While NMU and NMUR2 are highly conserved across species, their expression has high inter-animal variability (Gartlon et al., 2004), which may influence behaviors associated with substances of abuse. Individual differences in behavioral responses to drugs are thought to be partly mediated by genetic factors (Egervari et al., 2018). This has translational relevance as a single nucleotide polymorphism in NMUR2 is associated with alcoholism in humans (Johnson et al., 2006). Together, this suggests that basal inter-animal variability and cocaine-evoked changes in NMU and NMUR2 expression should be studied to determine their respective contributions to cocaine-evoked behaviors.

While the effects of NMU and NMUR2 on drug sensitization have been explored (Kasper et al., 2016), the effects of cocaine on NMU and NMUR2 expression remain unknown. We hypothesize that repeated cocaine exposure leads to dysregulated NMU and NMUR2 function in the context of cocaine-evoked locomotor activity. Here, we determine the effects of acute vs. sensitized cocaine exposure on NMU and NMUR2 expression in the NAc and correlate this expression to cocaine-evoked locomotor activity.

## Materials and Methods

### Animals

Male Sprague-Dawley rats (*n* = 42; Harlan, Houston, TX, USA) weighing 225 g to 250 g were singly housed, given *ad libitum* access to food and water in their home cages, and maintained on a 12–12 light-dark cycle. This study was carried out in accordance with the recommendations of the Guide for the Care and Use of Laboratory Animals and the approval of the University of Texas Medical Branch Institutional Animal Care and Use Committee. The protocol was approved by the University of Texas Medical Branch Institutional Animal Care and Use Committee.

### Cocaine Sensitization Model

Cocaine hydrochloride (NIDA, Washington, DC, USA) was prepared and rats were sensitized to cocaine using a method published previously (Filip et al., 2004; Kasper et al., 2016). Briefly, rats were divided into three groups (control, non-sensitized and sensitized with *n* = 14 per group) and received intraperitoneal (IP) injections of either 0.9% saline (control and non-sensitized) or 15 mg/kg cocaine hydrochloride (sensitized) twice per day, 8 h apart, for 5 days. Locomotor activity was measured for 1 h following first IP administration using an open field enclosure and photobeam matrix (San Diego Instruments, San Diego, CA, USA). Following 7 days of abstinence, all animals except control were given one IP injection of 15 mg/kg cocaine. Challenge day locomotor activity (total number of beam breaks) was analyzed using analysis of variance (ANOVA) and planned comparisons (control vs. non-sensitized and non-sensitized vs. sensitized). Animals were split into separate groups for mRNA and immunohistochemistry (IHC) analysis.

### RT-PCR

NAc expression of NMU and NMUR2 in both sensitized and non-sensitized animals was quantified by RT-PCR (7500 Fast Real Time PCR system, Applied Biosystems). Rats were euthanized with Ketamine/Xylazine (Benzon et al., 2014; McCue et al., 2018), and the NAc was microdissected on ice (Paxinos and Watson, 2007). NAc tissue was homogenized, RNA was extracted (74104, Qiagen, Germantown, MD, USA) and converted to cDNA (170-8891, BioRad, Hercules, CA, USA). NAc cDNA was amplified using the following primers: NMU mRNA and NMUR2 mRNA (NMU Rn00573761_m1, NMUR2 Rn00574015_m1, TaqMan, Applied Biosystems, Foster City, CA, USA). All samples were equally distributed across 96 well plates. C_T_ values were normalized to the housekeeping gene PPIA (Rn00690933_m1, TaqMan, Applied Biosystems, Foster City, CA, USA), and quantified for each sample. Differences in NMU and NMUR2 mRNA expression in non-sensitized and sensitized animals were normalized to control animals and converted to fold change.

### Immunohistochemistry

Accumbal NMUR2 was visualized in perfused brains using immunohistochemistry methods described previously (Benzon et al., 2014; Kasper et al., 2016; McCue et al., 2016). Briefly, 40 μm sections were taken from an anterior to posterior range of the NAc (three slices from each rat at 2.76, 1.92 and 1.32 mm from Bregma) to mimic the range included in the tissue dissections for RT-PCR. Slices were permeabilized, blocked with serum in phosphate buffered saline, and incubated with rabbit αNMUR2 (1:150; NBP-02351, Novus Biologicals). The sections were washed and incubated with donkey αrabbit AF-488 (1:100). Images were acquired using a Leica True Confocal Scanner SPE and Leica Application Suite Advanced Software (Leica Microsystems, Wetzlar, Germany) with 20× objective and tile scan mode.

Accumbal NMUR2 staining was quantified using FIJI (ImageJ). Image background was subtracted using rolling ball radius of 100 pixels. To quantify neuronal cell bodies and synapses, the range for desired size of events was set to >10 pixels. This quantifies the number of NMUR2 positive cell bodies and synapses as “events.” To analyze images, the NAc was defined from the rat brain atlas (Paxinos and Watson, 2007), and the number of NMUR2 positive neuronal events were quantified.

### Statistical Method

GPower 3.1 software was used to determine group size necessary for correlations and Prism 6 software (GraphPad Software Inc., La Jolla, CA, USA) was used for all other statistical analyses. Total locomotor activity, mRNA and IHC compared non-sensitized and sensitized cocaine groups through ANOVA and two-tailed unpaired *t*-tests. mRNA and IHC were also expressed as a correlation between non-sensitized and sensitized cocaine, and were analyzed for significance using Pearson’s *R-squared* test.

## Results

### Cocaine Sensitization Does Not Alter NMU mRNA Expression

**Figure 1:**
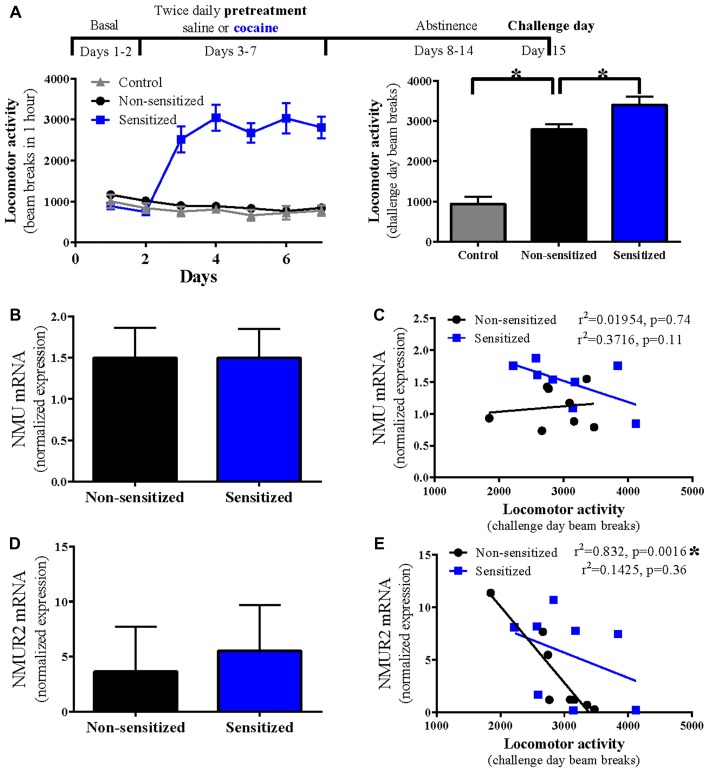
Cocaine sensitization disrupts correlation of accumbal neuromedin U receptor 2 (NMUR2) mRNA expression with cocaine-evoked locomotion. **(A)** The timeline and locomotor activity for cocaine sensitization paradigm. Locomotor activity was measured in animals given saline injection or cocaine injection twice daily for 5 days (*n* = 14 per group). After 7 days of abstinence, locomotor activity was measured for 1 h following injection on challenge day where control animals received saline and the other groups both received cocaine. **(B)** Neuropeptide NMU mRNA expression in the nucleus accumbens (NAc; *n* = 8) and **(C)** the correlation between NMU mRNA and locomotor activity. **(D)** NMUR2 mRNA expression in the NAc (*n* = 8) and **(E)** the correlation between NMUR2 mRNA and locomotor activity. Data shown as bar graphs are mean ± SEM. *p < 0.05 vs. non-sensitized. Data shown as correlations are individual subjects with correlation line fit to data. *p* values are reported on correlation figures with *p < 0.05.

Rats were successfully sensitized, with twice daily cocaine (15 mg/kg) for 5 days, to the locomotor effects of cocaine (sensitized) compared to control rats receiving acute cocaine (non-sensitized; *n* = 14 per group; *p* = 0.02 Figure 1A). No stereotyped behaviors or repeated beam breaks were observed that may interfere with locomotor activity assay. To determine the effect of cocaine sensitization on NMU mRNA expression, accumbal NMU mRNA was compared between non-sensitized and sensitized rats (Figure 1B) where no difference was observed (*n* = 8 per group; *p* = 0.99; Figure 1B). To examine the relationship between NMU mRNA expression and total cocaine-evoked locomotor activity, correlations were performed in each group but no significant correlation was found (non-sensitized *p* = 0.74; sensitized *p* = 0.11; Figure 1C).

### NMUR2 mRNA Negatively Correlates With Cocaine-Evoked Locomotion

Similar to NMU, we examined the effect of cocaine sensitization on accumbal NMUR2 mRNA expression and found no difference on total expression levels between non-sensitized and sensitized rats (*n* = 8 per group; *p* = 0.37; Figure 1D). The high standard error levels make it difficult to detect differences in total mRNA and indicate the individual differences for NMUR2 expression. In fact, some animals demonstrate a greater than 40 fold difference in NMUR2 mRNA levels. This prompted us to again evaluate the correlative relationship between NMUR2 mRNA and cocaine-evoked locomotor activity. When examining this relationship, we observed a significant negative correlation in non-sensitized rats. However, this correlation is absent in sensitized rats (non-sensitized *p* = 0.0016; sensitized *p* = 0.36; Figure 1E). Thus, repeated administration of cocaine disrupts the correlation between NMUR2 mRNA in the NAc and cocaine-evoked locomotor activity.

### NMUR2 Protein Negatively Correlates With Cocaine-Evoked Locomotion

**Figure 2:**
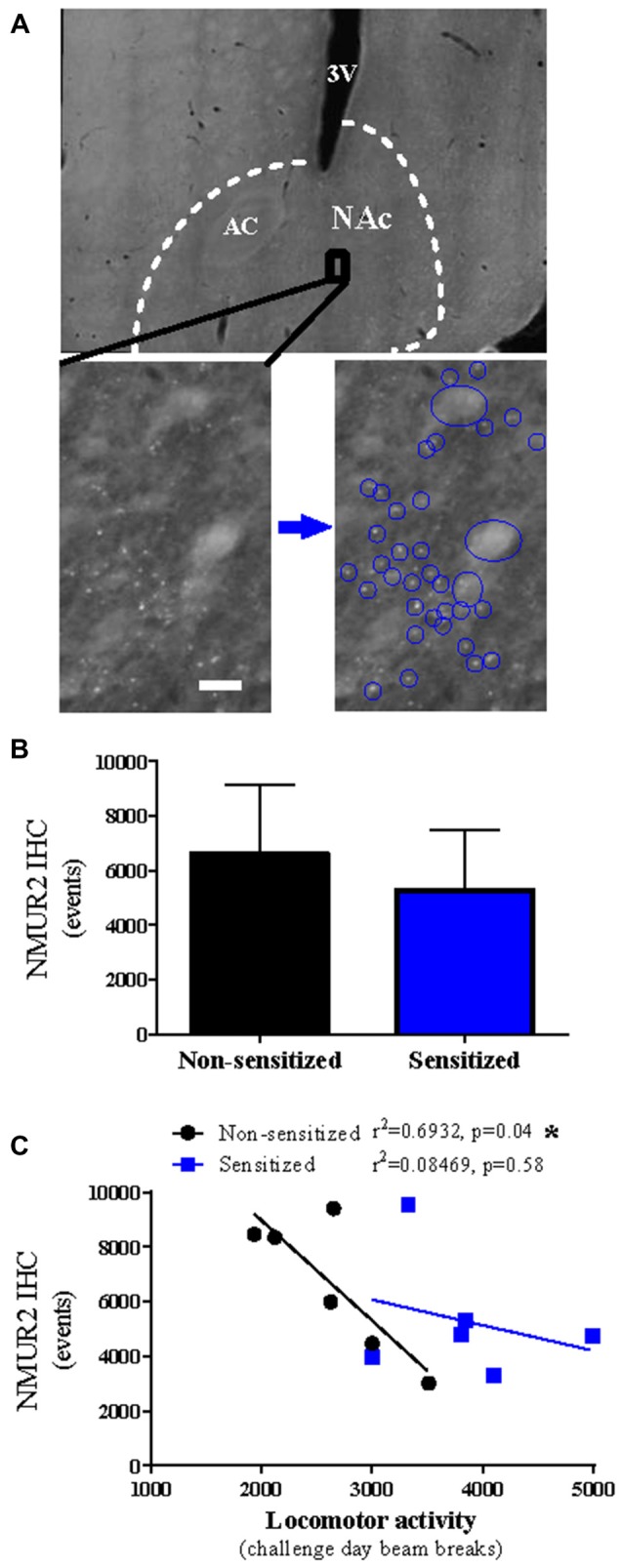
Cocaine sensitization disrupts correlation of accumbal NMUR2 protein expression with cocaine-evoked locomotion. **(A)** Representative immunohistochemistry (IHC) staining for NMUR2 in the NAc with white dashed lines surrounding the NAc and black box indicating the zoomed in area below. Staining patterns for large cell bodies and synaptic puncta can be observed (blue circles). 3V indicates third ventricle, AC indicates anterior commissure, and white bar indicates 10 μm. **(B)** The quantification of NMUR2 positive staining events (*n* = 6 per group) and **(C)** the correlation between the NMUR2 IHC events and locomotor activity. Data shown as bar graphs are mean ± SEM. Data shown as correlations are individual subjects with correlation line fit to data. *p* values are reported on correlation figures with *p < 0.05.

To determine if the observed mRNA changes in NMUR2 expression between sensitized and non-sensitized rats effected NMUR2 protein, IHC for NMUR2 was performed to visualize and quantify the NMUR2 positive cell bodies and synapses. Confirming previous studies (Kasper et al., 2016), NMUR2 protein staining is enriched in the NAc and was expressed on cell bodies and in a “beads on a string” motif (Figure 2A). To determine if NMUR2 staining pattern is altered following cocaine sensitization, the total numbers of discrete NMUR2 staining events were quantified. There was no difference between non-sensitized and sensitized rats (*n* = 6 per group; *p* = 0.35; Figure 2B). However, examining the relationship between NMUR2 staining and cocaine-evoked locomotion, there was a negative correlation between NMUR2 protein staining events and cocaine-evoked locomotor activity in rats receiving acute cocaine (non-sensitized) that was absent in rats receiving chronic cocaine (sensitized; non-sensitized *p* = 0.04; sensitized *p* = 0.58; Figure 2C). Similar to what was observed with NMUR2 mRNA, repeated administration of cocaine disrupts the correlation between NMUR2 protein and cocaine-evoked locomotor activity.

## Discussion

This study elucidates the relationship between cocaine exposure and accumbal NMU and NMUR2. Both NMU and NMUR2 modulate alcohol intake (Vallöf et al., 2017), amphetamine-evoked locomotion (Vallöf et al., 2016), and cocaine sensitization (Kasper et al., 2016), but it was unclear if exposure to substances of abuse modulates NMU or NMUR2. Additionally, it is unknown if the high inter-animal variability in NMU and NMUR2 (Gartlon et al., 2004) influences behavioral responses to cocaine. Here, in non-sensitized rats receiving cocaine for the first time on challenge day, we observed a significant negative correlation between cocaine-evoked locomotion and NMUR2 mRNA and protein expression. However, in cocaine-sensitized rats, the correlation was lost. These data indicate that repeated cocaine leads to a dysregulation of NMUR2 presence in the context of cocaine-evoked activity and suggest that normal NMUR2 signaling acts as a behavioral brake on cocaine-evoked locomotor activity. Thus, repeated cocaine exposure may remove this behavioral brake by causing dysregulated NMUR2 expression in the NAc resulting in increased locomotor activity to cocaine ultimately promoting behavioral sensitization.

The lack of correlation between NMU and cocaine-evoked locomotion (Figure 1C) was unexpected as intracerebroventricular NMU decreases non-sensitized amphetamine-evoked activity (Vallöf et al., 2016), and intra-NAc NMU decreases non-sensitized cocaine-evoked activity (Kasper et al., 2016). It is possible that the concentration of NMU necessary to alter behavior is greater than the endogenous inter-animal range of NMU expression. This suggests that NMUR2 agonists previously studied for obesity (Sampson et al., 2018) may suppress cocaine-evoked behavior. The repeated saline injections may also influence NMU and NMUR2, or could cause stress-induced changes which limit some interpretation in this study.

The negative correlation of NMUR2 with acute cocaine-evoked locomotion is consistent with previous studies indicating that knockdown of NAc NMUR2 increases cocaine-evoked locomotion (Kasper et al., 2016). Additionally, previous work demonstrates intra-NAc NMU does not alter cocaine-evoked locomotion when administered to sensitized rats before cocaine challenge day (Kasper et al., 2016), which complements the loss of correlation in sensitized rats of NMUR2 with cocaine-evoked locomotion. However, the correlation presented here does not demonstrate a direct link and the functional relevance of NMUR2 during repeated cocaine exposure is an ongoing area of research.

Together, these results extend the previous studies on NMUR2 in modulating behaviors related to substances of abuse (Kasper et al., 2016; Vallöf et al., 2016, 2017) by adding the first evidence that a substance of abuse influences the role of endogenous NMUR2 in behavior. These data also demonstrate the inter-animal variability in NMU and NMUR2 expression specifically in the NAc and suggests this variability may contribute to individual differences in initial behavioral responses to cocaine. Restoring the relationship between accumbal NMUR2 and behavioral responses to cocaine may have therapeutic value, and NMUR2 remains a promising pharmacotherapeutic target.

## Author Contributions

JK and JH designed the study. JK performed the experiments. JK and AS analyzed the data. All authors have contributed and approved the manuscript.

## Conflict of Interest Statement

The authors declare that the research was conducted in the absence of any commercial or financial relationships that could be construed as a potential conflict of interest.
